# Anxiety, coping style and hopelessness during COVID‐19 pandemic: An Iranian population‐based study

**DOI:** 10.1002/hsr2.1233

**Published:** 2023-05-02

**Authors:** Khodamorad Momeni, Yahya Salimi, Mohammad Reza Majzoobi, Arash Ziapour, Parisa Janjani

**Affiliations:** ^1^ Faculty of Social Sciences Razi University Kermanshah Iran; ^2^ Social Development & Health Promotion Research Center, Health Institute Kermanshah University of Medical Sciences Kermanshah Iran; ^3^ Department of Epidemiology, School of Health Kermanshah University of Medical Sciences Kermanshah Iran; ^4^ Institute of Psychology Siegen University Westphalia Germany; ^5^ Cardiovascular Research Center, Health Institute, Imam‐Ali hospital Kermanshah University of Medical Sciences Kermanshah Iran

**Keywords:** anxiety, coping style, COVID‐19, hopelessness, Iran, pandemic

## Abstract

**Background and Aims:**

The COVID‐19 pandemic has caused new conditions such as nationwide quarantine, a dramatic decrease in‐person interaction and an increase in death anxiety for governments and people. The pandemic of an unpredictable disease with no definite treatment can pose physical and psychological risks to individuals. The present study aimed to investigate the state of anxiety, coping styles, and hopelessness of people in the lockdown period and reopening in Iran.

**Methods:**

In this national population‐based cross‐sectional study, a total of 1191 people who had access to social networks from all over Iran completed the anxiety, hopelessness, and coping style questionnaires online using the snowball sampling method. Analysis was conducted using Stata software version 12 (Stat Corp). The significance level was set at 0.05.

**Results:**

The results of the study suggested that women experienced higher levels of anxiety than men, and, in stressful situations, women were more likely to use emotion‐focused coping styles, while men frequently used problem‐focused coping styles. The majority of participants reported moderate levels of anxiety and low levels of hopelessness (64.04%). The results of multiple linear regression show any level of anxiety mild (*ß* = 0.59, 95% confidence interval [CI]: 0.32–0.85), moderate (*ß* = 0.72, 95% CI: 1.36–1.08), and severe (*ß* = 2, 95% CI: 1.36–2.56) relative to the none anxiety significantly increase the hopelessness, Furthermore, showing a negative significant adjusted association with the problem‐focused coping style (*ß* = −0.06, 95% CI: −0.07 to −0.04), and a significant positive association with the emotion‐focused coping style (*ß* = 0.04, 95% CI: 0.02–0.06).

**Conclusion:**

Our findings could be used to prevent psychological damage in societies and suggested addressing problem‐focused coping style, especially during a crisis, and providing people with preprepared mental health protocols at this pandemic.

## 1 INTRODUCTION

The pandemic of severe acute respiratory syndrome coronavirus 2 (SARS‐CoV‐2) in late December 2019 has caused global public health to face significant challenges and led people to experience unpleasant feelings worldwide.^1^ Iran is one of the countries that faced the outbreak of coronavirus immediately after China. On February 19, 2020, the Iranian Ministry of Health officially announced that two people in Qom were infected with the coronavirus. Meanwhile, it spread dramatically in Gilan, Tehran and almost all over Iran.^1^, ^2^ According to the Corona National Committee (21 July), 278,827 people were infected with this virus, and 14,634 people lost their lives.^3^

In addition to physical harm, the disease also appears to cause psychological damage. One of the psychological symptoms of any unknown disease and this disease is anxiety.^4^, ^5^, ^6^ Low scientific knowledge, and unknown and unpredictable consequences of the virus caused distress and anxiety in people around the world.^7^ Anxiety is a common response to stressful situations, and the experience of anxiety about coronavirus disease 2019 (COVID‐19) is widely reported. Another psychological effect of coronavirus on people is experiencing feelings of hopelessness and worry about the future. The unpredictability of the virus's behavior made people feel helpless, and, as a result, people found a negative attitude toward themselves, the world, and the future.^8^ Studies showed that the spread of SARS‐CoV‐2 affected people's mental health.^9^ Roy et al.^10^ examined the psychological problems of people with the breakout of corona in China and assessed the mental health status of 1074 Chinese. Their study suggested higher levels of anxiety, depression, and alcohol consumption which were recognized as dangerous and harmful and also lower levels of mental well‐being than normal. They also reported that young people between the ages of 21 and 40 were more vulnerable to mental health problems.^10^ Moghanibashi‐Mansourieh^11^ studied 10,754 Iranians and showed that at the time of the corona outbreak, people aged 20–40 and those who followed the news on corona were more anxious. Women also had higher anxiety levels than men, and people who experienced Coronavirus infection in their family reported higher anxiety levels.^12^ Investigating 662 Indians, Roy et al.^10^ showed that the level of public awareness about the coronavirus was moderate, and the level of anxiety of the participants in the study was high. In such a crisis, people take steps to reduce the effects of stress in the face of stressful cases and to control the situation, which determines the set of coping styles in a crisis.^10^ In the beginning of a stressful event, human beings use problem‐focused and emotion‐focused coping styles. Each styles can be effective or troublesome depending on the type of stress, and the situation faced.^13^ So far, the coping styles of individuals in such critical cases have never been studied, which has provided the basis for appropriate psychological interventions.

Scientific evidence shows that most of the studies conducted during the COVID‐19 pandemic investigated health, nutritional, vaccine, etc. issues. While psychological issues and coping styles are also important in critical situations. By identifying people's coping style in critical situations and timely training, people can be psychologically vaccinated to bear less anxiety and stress. Considering the importance of the subject and the limited studies in this field, despite the existence of several crises such as earthquakes, floods, diseases, etc., the present study aims to investigate the state of anxiety, hopelessness, and coping styles of individuals.

## 2 METHODS

### 2.1 Study design and participant recruitment

It was a national population‐based cross‐sectional study conducted during the pandemic of COVID‐19 in Iran between May 01, 2020 and May 13, 2020. All Iranian adults from 31 Provinces of Iran were considered as the statistical population. Based on similar studies that were conducted online and regarding COVID‐19 pandemic, the study samples estimated about 1000 people.^9^, ^11^ Due to the reluctance of some people to participate in the study and answer the questions, the questionnaire was sent to at least 2500 people, to reached the desired sample size. Finally, 1191 subjects who completed the questionnaire were included in the present study.

All subjects voluntarily participated and were identified by snowball sampling through communication channels and social media. Participants with incomplete data (such as the name of the province or age, etc.) were excluded from the study. The study participants comprised 1191 adults considering the following inclusion criteria: age over 16 years, living in Iran. Inform consent was obtained at the start of an online survey. This study has not been done according to the Checklist for Reporting Results of Internet E‐surveys (CHERRIES). Still, things like (the informed consent page/Number of Items/Number of screens/a Back button or a Review step which displays a summary of the responses and asks the respondents if they are correct/) in the checklist have been observed. The Ethical Committee of the Kermanshah University of Medical Sciences approved the study with reference number: IR.KUMS.REC.1399.1105. Written informed consent was obtained from all participants, and emphasized on the confidentiality of personal information. All procedures performed in studies involving human participants were in accordance with the ethical standards of the institutional research committee and with the 1964 Helsinki Declaration and its later amendments or comparable ethical standards.

### 2.2 Measures

The first section of the instrument contained the demographics (Age, Gender, Marital status, Educational level, Job title, Income (per month), Impact of the COVID‐19 pandemic on job and income, The way of pandemic impact on job and income and the History of chronic disease). In the present study, all participants completed an online questionnaire that included Beck Anxiety Inventory (BAI^14^); Iranian version^15^, Beck Hopelessness Scale (BHI^16^); Iranian version^13^, ^17^; and Iranian version.^18^

### 2.3 BAI

BAI is a 21‐item screening tool for assessing anxiety symptoms with intensity. This inventory assesses the emotional, physiological, and cognitive symptoms of anxiety. Participants are asked to rate the degree to which each item troubled them over the past 7 days on a 4‐point scale ranging from 0 (“not at all”) to 3 (“severely ‐ I could barely stand it”). The attainable range of BAI can be varied between 0 and 63. The authors recorded the BAI's alpha (0.92) of high Cronbach and strong test‐retest reliability (0.75) for a 1‐week gap.^19^ This scale had a moderately high correlation (0.51) with the Hamilton Anxiety Rating Scale.^19^ Five types of content validity, simultaneous, diagnostic construct and complete, have been measured for this test, which all show the high efficiency of this tool in measuring the intensity of anxiety.^16^ The anxiety score was categorized as minimal anxiety levels (0–7), mild anxiety (8–15), moderate anxiety (16–25), and severe anxiety (26–63).^20^

### 2.4 BHI

In 1974, Beck et al.^16^ designed the hopelessness questionnaire to measure hopelessness at the cognitive therapy center of the University of Pennsylvania. To investigate its psychometric properties, they implemented it for various samples of patients.^16^

BHI rates 20 items on a 6‐point Likert scale (from “1” entirely disagree to “6” completely agree) to measure hopelessness. Beck Hopelessness Scale was developed by Beck et al.^16^ in the UK. Its adaptation to Iran was undertaken by Dejkam.^17^ Each question had a score of 1 or 0. Of the 20 questions, 9 were false, and 11 were true answers. The score scale ranges between 0 and 20, such that score in the 0‐3 range is the minimum sense of hopelessness, 4–8 mild hopelessness, 9–14 moderate hopelessness and over 14 severe degrees of hopelessness.^17^

Dejkam^17^ reported the high validity of this test in cancer patients (*r* = 0.8), which seems that this test can be used as a suitable tool for evaluating pessimistic attitudes and hopelessness with valid psychometric properties. Applying the test in Iran, it has a Cronbach's *α* of 0.75, which indicates good internal consistency of the test.^17^

### 2.5 Coping styles questionnaire (WCQ)

This questionnaire has 66 questions based on the list of coping strategies of Lazarus and Folkman.^13^ It evaluates a wide range of thoughts and actions that people use when facing internal or external stressful conditions.^13^ Its scoring is based on the Likert scale from 0 to 3.^12^, ^13^ This questionnaire has 8 subscales of coping, distancing, self‐control, seeking social support, accepting responsibility, avoiding avoidance, planned problem‐solving and positive reappraisal. Sixteen questions of this questionnaire are deviant, and the other 50 questions evaluate the person's coping style. The validity of this questionnaire was tested in a sample of 750 middle‐aged couples. Cronbach's *α* coefficient of direct confrontation was 0.70, distancing at 0.61, self‐control 0.71, seeking social support 0.76, and acceptance of responsibility 0.66. Avoidance was reported at 0.72, planned problem solving at 0.67, and positive reappraisal at 0.79.^8^

### 2.6 Statistical analysis

Analysis was conducted using Stata software version 12 (Stat Corp). The significance level was set at 0.05. The independent *t*‐test, chi‐square and Fisher exact two‐sided test were used in the bivariate analysis. Multiple linear regression models with hopelessness as the dependent variable were used to estimate adjusted associations with anxiety and coping styles. Several variables were examined to detect their potential confounding role, including age, gender, marital status, educational level, job title, the impact of COVID‐19 pandemic on job and income and history of chronic disease.

## 3 RESULT

Participants’ age ranged from 16 to 76 years. The mean (Standard Deviation) age for women and men were 32.79 (11.07) and 31.28 (10.94) years, respectively. Also, the majority of participants were in the age group 18 to 40 years old (0.80%). The demographic characteristics of participants are presented in Table 1.

**Table 1:** Socio‐demographic characteristics of Participants (*n* = 1191).

Variable		Gender	
		Male	Female
		*N* (%)	*N* (%)
Age groups	18–40 years	104 (23.20)	345 (76.80)
	40–60 years	107 (28.80)	264 (71.20)
	Over 60 Years	114 (30.70)	257 (69.30)
Marital status	Single	156 (24.80)	472 (75.20)
	Married	165 (30.60)	375 (69.40)
	Divorced	4 (17.40)	19 (82.60)
Educational level	Less than a diploma	15 (35.70)	27 (64.30)
	Diploma or postgraduate diploma	77 (22.90)	259 (77.10)
	Bachelor's & higher	233 (28.70)	580 (71.30)
Job title	Government official	114 (33.00)	231 (67.00)
	Self‐employed	85 (45.70)	101 (54.30)
	Retrieved	14 (48.30)	15 (51.70)
	Student	87 (19.60)	357 (80.40)
	Housewife	1 (0.70)	134 (99.30)
	Unemployed	24 (46.20)	28 (53.80)
Income (per month)	None	68 (16.50)	343 (83.50)
	Less than 50 $	64 (28.80)	158 (71.20)
	Between 50 and 150 $	58 (31.70)	125 (68.30)
	Between 150 and 250 $	111 (34.80)	208 (65.20)
	Between 250 and 350 $	16 (38.10)	26 (61.90)
	Over 350 $	8 (157.10)	6 (42.90)
Impact of COVID‐19 pandemic on job and income	Yes	156 (32.50)	324 (67.50)
	No	169 (23.80)	542 (76.20)
The way of pandemic impact on job and income	Increase in income	3 (13.60)	19 (86.40)
	Decrease in income	81 (25.10)	242 (74.90)
	Losing a job but receiving unemployment benefits	0 (0.0)	9 (100.0)
	Losing a job without getting paid	40 (32.30)	84 (67.70)
History of chronic disease	Yes	31 (24.60)	95 (75.40)
	No	294 (27.60)	771(72.40)

The mean (SD) score of anxiety was significantly higher in women compared to men, 6.28 (7.87) for men and 8.91 (8.26) for women (*p* < 0.001). A significant difference was observed in coping styles. Men significantly selected problem‐focused coping (*p* = 0.01), whereas women were more likely to choose emotion‐focused coping (*p* = 0.01) (Table 2).

**Table 2:** Gender‐based differences in anxiety, hopelessness, and ways of coping after the COVID‐19 epidemic (*n* = 1191).

Variable	Men (*N* = 325)	Women (*N* = 866)	*t* statistics (*p*‐value)	Effect sizes
	Mean (SD)	Mean (SD)		
Anxiety	6.28 (7.87)	8.91 (8.26)	−4.94 (<0.001)	−0.32
Hopelessness	8.35 (2.15)	8.30 (2.09)	0.40 (0.68)	0.03
Coping styles				
Problem‐focused coping	37.94 (11.85)	36.84 (10.03)	1.60 (0.11)	0.19
Emotion‐focused coping	36.96 (9.86)	38.33 (8.48)	−2.38 (<0.05)	0.008

Abbreviations: COVID‐19, coronavirus disease 2019; SD, standard deviation.

The results showed that about 41% of participants reported suffering from different anxiety levels (mild 25.36%, moderate 11.75%, and severe 4.28%) during the COVID‐19 outbreak. Likewise, most had different levels of hopelessness (mild 64.04%, moderate 34.78%, and severe 1.10%).

A significant difference in anxiety levels (*χ* ^2^ = 18.99, *p* < 0.001) was observed between people affected by COVID‐19 and people who did not. Frequencies of moderate and severe anxiety were 111 (13.47%) and 27(3.27%), respectively, for people affected with COVID‐19, while they were 29 (8.35%) and 24 (6.91%) for none involved with COVID‐19 (Table 3).

**Table 3:** Differences in scores of anxiety and hopelessness in those who were infected with COVID‐19 versus those who had no infection (*n* = 1191).

Variable	People none contracted with the COVID‐19	People contracted with the COVID‐19	*χ* ^2^ (*p*‐value)
	*N* (%)	*N* (%)	
Anxiety			18.99 (<0.001)
None	495 (0.60)	203 (0.58)	
Mild	191 (23.17)	111 (31.98)	
Moderate	111 (13.47)	29 (8.35)	
Severe	27 (3.27)	24 (6.91)	
Hopelessness			2.35 (0.50)
Mild	526 (64.0)	229 (63.0)	
Moderate	282 (34.0)	128 (35.0)	
Severe	9 (1.0)	4 (1.0)	

Abbreviation: COVID‐19, coronavirus disease 2019.

Table 4 shows the results of multiple linear regression with a history of incidence of COVID‐19 in the network, anxiety level, problem‐focused coping style and emotion‐focused coping style as the independent variable and hopelessness as the outcome. The model adjusted for socio‐demographic factors (age, gender, place of living, marital status, educational level, income level, and history of chronic disease) showed a history of incidence COVID‐19 in‐network non‐statistically significant association with hopelessness (*ß* = −0.10, *p* = 0.44). The adjusted associations between higher anxiety levels and hopelessness were statistically significant. Importantly, mild (*ß* = 0.59, 95% CI: 0.32–0.85), moderate (*ß* = 0.72, 95% CI: 1.36–1.08), and severe anxiety levels severe (*ß* = 2, 95% CI: 1.36–2.56) compared with none anxiety appeared to experience more hopelessness. Also, hopelessness showed a negative significant adjusted association with the problem‐focused coping style (*ß* = −0.06, *p* < 0.001) and a significant positive association with the emotion‐focused coping style (*ß* = 0.04, *p* < 0.001).

**Table 4:** Multiple linear regression results of variables associated with hopelessness in Iranian adults, 2020 (*n* = 1191).

Variables	*ß*	95% confidence Interval	
Age	0.008	−0.005	0.02
Female gender	−0.37	−0.64	−0.10
Place of living			
Urban	Ref	ref	ref
Rural	−0.15	−0.60	0.28
Marital status			
Single	Ref	ref	ref
Married	−0.37	−0.66	−0.08
Divorced	−0.37	−1.09	0.59
Educational level			
Less than a diploma	Ref	ref	ref
Diploma or postgraduate diploma	−0.87	−1.52	−0.23
Bachelor's & higher	−0.97	−1.62	−0.33
Job Title			
Governmental employee	Ref	ref	ref
Nongovernmental employee	0.18	−0.20	0.57
Retired	0.70	−0.09	1.50
Student	0.40	−0.04	0.58
Housekeeper	0.47	−0.03	0.98
Unemployed	0.55	−0.10	1.21
Have a history of chronic disease	0.51	0.14	0.89
Income (per month)			
No income	Ref	ref	ref
Less than one million	0.29	−0.60	0.65
Between one to three million	0.16	−0.26	0.59
Between three to five million	0.17	−0.26	0.61
Between five to seven million	0.29	−0.39	0.98
Over seven million	−0.32	−1.41	0.76
History of incidence COVID‐19 in network	−0.10	−0.34	0.14
Anxiety level			
None	Ref	ref	ref
Mild	0.59	0.32	0.85
Moderate	0.72	0.36	1.08
Sever	2.00	1.43	2.56
Problem‐focused coping style	−0.06	−0.07	−0.04
Emotion‐focused coping style	0.04	0.02	0.06

*Note*: Numbers in bold are significant at 0.05 level.

## 4 DISCUSSION

New SARS‐CoV‐2, which was followed by severe physical consequences, has surprised public health services all over the world. Various studies have been conducted during the time of Covid‐19.^21^, ^22^, ^23^, ^24^

But its psychological consequences in the general population in the west of Iran were somewhat neglected or at least not prioritized, and most of the study's psychological consequences are on recovered people who have experienced covid.^25^, ^26^ Therefore, the research literature on the general population is limited. The present study looked at people's anxiety, hopelessness, and coping styles during the coronavirus outbreak, three months after its outbreak, while, naturally, the severity of the crisis reduces over time, despite its persistence.

In the present study, people reported lower levels of anxiety. Conducted in March in Iran, Moghanibashi‐Mansourieh (2020) showed higher levels of anxiety among individuals.^12^ In China, Ahmed et al.^9^ also reported high levels of participants’ anxiety. Public health measures, such as avoiding social environments, to prevent the disease make people feel isolated and lonely, which may increase stress and anxiety. One of the reasons for the decrease in anxiety in the present study was finishing the lockdown and returning to the social environment and society.^9^ Gradual reopening of the economy, accepting the disease psychologically, adapting to the prevailing conditions due to the uncertainty of its end, the transition from the quarantine period and severe restrictions in the current situation were helpful. In addition, the prevalence of the disease in Iran declined during the study, and compared to previous months, the national media reported positive news to the public.^27^ Considering the above, it is evident that compared to the first months of the coronavirus outbreak in Iran, we may face a relative decrease in the experience of anxiety.

Other results from the current study indicated that women were more likely to be anxious than men. These findings are consistent with that of Moghanibashi‐Mansourieh.^12^ However, in China, men's anxiety was reported to be higher than that of women.^9^ The findings could be interpreted as higher spirit of support and attachment to family members in women compared to men, and, therefore, the fear of losing loved ones in crises made women more annoyed compared with men and caused them more anxiety. On the other hand, financial resources for women are more limited compared to men, which can increase the level of anxiety. Some studies also showed that death anxiety was higher among women than men.^28^, ^29^ For social‐cultural reasons, it seems, in Iranian society, women were more influenced by rumors than men. In critical situations such as the Coronavirus outbreak, women were more likely to accept stories about dangers. Women were found to use social networks much wider than men in Iran. A large amount of fake information was introduced on these networks, and bad news was reported to be more prevalent among women. Therefore, these factors may help women experience more anxiety.

The results of the present study showed that women in crises use an emotion‐focused coping style, and most men use a problem‐focused coping style to manage stress in stressful and critical situations. Using healthy coping styles in facing stress can be effective in self‐care behavior. Looking at the relationship between anxiety and coping styles in terms of cancer as a stressful event Perez‐Ordonez et al.^30^ suggested a relationship between anxiety and emotion‐focused coping style in traumatic situations. In the present study, women reported higher anxiety, and it made sense to use an emotional coping style to manage stress in crisis situations.

Other study results showed that people experienced different levels of hopelessness. Most of the people in this study reported low and moderate levels of hopelessness. No study on coronavirus outbreak is available to date. Still, the results from Pourmovahed et al. in an Iranian city reported a significant relationship between increasing age and hopelessness, and the older people are, the more frustrated they feel. Compared with the present study, most of the participants in their study educated young adults with a higher level of awareness and were more hopeful about the future and medical science to find a way to prevent and treat the disease.^31^ The World Health Organization (WHO) stated that coronavirus would affect most older people and that the death rate was higher among the elderly.^1^ Based on this evidence, given that most participants in the present study were young adults, people's age was an essential factor in reporting relatively low and moderate hopelessness. It is worth noting that the role of registering preventive factors, such as setting up free lines to receive psychological services in Iran, was not ineffective. In this program, people requiring psychological services used the services on the phone. The Corona National Committee also managed to reduce the level of anxiety to some extent by launching a screening and identification system for people at risk, and people felt that the Ministry of Health supported them. The Iranian government also announced through the national media that the situation was under control and that there were no concerns about hospital equipment and sanitary facilities.^32^

Findings from the present study reported that people who were infected with coronavirus or those who witnessed their relatives suffering from the disease experienced a higher level of anxiety than those who were not affected by a coronavirus. The results of the present study were consistent with the findings from Afrashteh and Janjani.^12^ People who were infected by coronavirus or witnessed the experience of the disease (e.g., severe breathing symptoms, body pain, the possible experience of going into a coma) in their relatives were fully aware of the danger of infection and found themselves more fearful and anxious due to the unpredictability of the coronavirus. However, people who did not have such an experience may see the disease as far away from them or think that they themselves may not suffer from the disease, thus experiencing less anxiety.

The results obtained from the regression model showed a significantly adjusted association between any level of anxiety (low, medium and high) and hopelessness. Although similar studies were not available in critical situations, the research literature and the results of other studies confirmed the present study's findings and were consistent with them.^33^, ^34^, ^35^ Their study also found a strong association between anxiety, hopelessness and depression. Considering the relatively rare diseases that affect about 3% to 4% of the world's population as a cause of stress and anxiety and a personal crisis, evidence suggested a relationship between anxiety and hopelessness in patients with vitiligo.^36^ At the same time, women reported higher levels of anxiety.^37^

Other results from the present study reported a negative relationship between Problem‐focused coping style and hopelessness in critical situations. This means that increasing the use of a Problem‐focused coping style reduces hopelessness. There was also a positive relationship between emotion‐focused coping style and hopelessness coping styles. People using an emotion‐oriented style more often were more likely to experience hopelessness. Since no studies were conducted on the relationship between coping styles and hopelessness during the pandemic, the results from the present study were compared to those from cancer studies. Cancer is also a serious physical illness that puts a tremendous psychological burden on a person and can be a personal crisis in a person's life.^25^ The results suggested a relationship between the level of hopelessness with stress and coping style in people with cancer. In this study, people with an inconsistent and exciting coping style also experienced more hopelessness and stress.^38^, ^39^

### 4.1 Limitations

Although the present study reported several significant findings, it included some limitations. Given society's current state, it was impossible for individuals to complete a face‐to‐face questionnaire, so the questionnaires were implemented online. People who accessed social networks were included in the study, and those with no access to social networks for any reason were omitted from this study. Moreover, because of the study's cross‐sectional design, it was impossible to infer causality between anxiety, coping styles and hopelessness. On the other hand, most studies on COVID‐19 were conducted at the beginning of the pandemic with a high level of confusion and anxiety about the virus. Therefore, the results of the present study made it possible to compare the psychological state of individuals over time in critical conditions as a continuum.

## 5 CONCLUSION

The present study aimed to investigate anxiety, hopelessness, and coping styles with the coronavirus outbreak. The results of the study suggested that women experienced higher levels of anxiety. We also found that people experienced relatively less anxiety compared to the results of previous studies conducted at the beginning of the coronavirus outbreak. Feelings of disappointment were reported low, while people confronting coronavirus in any form reported higher anxiety. Our study showed a relationship between anxiety and hopelessness at all levels. In the present study, women were likelier to use an emotion‐oriented coping style. There was also a relationship between coping styles and hopelessness. Living in lockdown conditions and imposing restrictions on a person seemed to impose additional anxiety. It seems necessary for the general public to implement a multifaceted approach at the personal, social and international levels. The following two recommendations are suggested to prevent and control psychological problems. 1) In crises, governments should teach problem‐solving coping skills so that people can manage the level of anxiety and, consequently, the hopelessness caused by the crisis. 2) Report on the psychological status of communities should be considered. Based on that, governments should develop guidelines and protocols for multifaceted psychological interventions at different levels, considering the cultural and social differences that may be used in future crises to minimize harm.

## AUTHOR CONTRIBUTIONS

**Khodamorad Momeni**: Conceptualization. **Yahya Salimi**: Data curation; formal analysis. **Mohammad Reza Majzoobi**: Investigation; methodology. **Arash Ziapour**: Data curation; resources; validation; writing—original draft; writing—review & editing. **Parisa Janjani**: Conceptualization; funding acquisition; investigation; supervision; writing—original draft; writing—review & editing.

## CONFLICT OF INTEREST STATEMENT

The authors declare no conflicts of interest.

## ETHICS STATEMENT

The purpose of this research was completely explained to the participants through the cover page of the questionnaire, and they were assured that their information would be kept confidential by the researcher. Informed consent from the participants was acquired as they agreed to participate in the study by reviewing the questionnaire's cover page and clicking on the provided link. Furthermore, for participants younger than 18 years of age, the participant was asked for the consent of the parent or guardian.

## TRANSPARENCY STATEMENT

The lead author Parisa Janjani affirms that this manuscript is an honest, accurate, and transparent account of the study being reported; that no important aspects of the study have been omitted; and that any discrepancies from the study as planned (and, if relevant, registered) have been explained.

## Data Availability

The data sets used in the study are available from the corresponding author upon reasonable request.
